# A Reliable Indirect ELISA Protocol for Detection of Human Antibodies Directed to SARS-CoV-2 NP Protein

**DOI:** 10.3390/diagnostics11050825

**Published:** 2021-05-02

**Authors:** Arwa A. Faizo, Thamir A. Alandijany, Ayman T. Abbas, Sayed S. Sohrab, Sherif A. El-Kafrawy, Ahmed M. Tolah, Ahmed M. Hassan, Esam I. Azhar

**Affiliations:** 1 Special Infectious Agents Unit, King Fahd Medical Research Center, King Abdulaziz University, P.O. Box 128442, Jeddah 21362, Saudi Arabia; aafaizo@kau.edu.sa (A.A.F.); atabdalhadi@kau.edu.sa (A.T.A.); ssohrab@kau.edu.sa (S.S.S.); saelkfrawy@kau.edu.sa (S.A.E.-K.); atoulah@kau.edu.sa (A.M.T.); hmsahmed@kau.edu.sa (A.M.H.); 2 Department of Medical Laboratory Technology, Faculty of Applied Medical Sciences, King Abdulaziz University, P.O. Box 80324, Jeddah 21589, Saudi Arabia

**Keywords:** COVID-19, SARS-CoV-2, ELISA, serology, seroprevalence, nucleocapsid

## Abstract

A few months ago, the availability of a reliable and cost-effective testing capacity for COVID-19 was a concern for many countries. With the emergence and circulation of new SARS-CoV-2 variants, another layer of challenge can be added for COVID-19 testing at both molecular and serological levels. This is particularly important for the available tests principally designed to target the S gene/protein where multiple mutations have been reported. Herein, the SARS-CoV-2 NP recombinant protein was utilized to develop a simple and reliable COVID-19 NP human IgG ELISA. The optimized protocol was validated against a micro-neutralization (MN) assay, in-house S-based ELISA, and commercial chemiluminescence immunoassay (CLIA). The developed assay provides 100% sensitivity, 98.9% specificity, 98.9% agreement, and high overall accuracy with an area under curve equal to 0.9998 ± 0.0002 with a 95% confidence interval of 0.99 to 1.00. The optical density values of positive samples significantly correlated with their corresponding MN titers. The assay specifically detects IgG antibodies to the SARS-CoV-2 NP protein and does not cross-detect IgG to the viral S protein. Moreover, it does not cross-react with antibodies related to other coronaviruses (e.g., the Middle East respiratory syndrome coronavirus or human coronavirus HKU1). The availability of this reliable COVID-19 NP IgG ELISA protocol is highly valuable for its diagnostic and epidemiological applications.

## 1. Introduction

It has been over a year since COVID-19 was declared a pandemic. SARS-CoV-2, the leading cause of COVID-19, belongs to the β-coronaviruses [1,2]. Its nucleocapsid (NP) comprises the nucleocapsid protein and a positive-sense single-stranded RNA [2,3]. The viral nucleocapsid is encased within phospholipid bilayers that contain the membrane, envelope, hemagglutinin-esterase, and spike (S) proteins [2,3]. The mucosal epithelium of the upper respiratory tract is the primary site of viral replication [1,4,5]. However, the virus can also replicate in several organs including the lower respiratory tract (e.g., lung and bronchus), kidneys, and stomach because of the broad expression of its cellular receptor angiotensin-converting enzyme 2 (ACE2) [6,7,8,9,10]. During the infection, the S protein is cleaved into S1 and S2 subunits that are responsible for binding to ACE2 and membrane fusion, respectively [11,12]. Upon viral entry into target cells, the viral transcription, translation, and genome replication, virion assembly and maturation take place, leading to the production of infectious progeny virions.

Over the last few months, scientists conducted a tremendous amount of research addressing COVID-19 virology, epidemiology, evolution, diagnosis, vaccines, and antivirals. Serology tests are valuable tools and highly applicable techniques in these research areas [13]. Indeed, serology tests complement molecular techniques for diagnosis purposes (e.g., diagnosis of asymptomatic patients) and epidemiolocal applications (e.g., seroprevalence studies) [13]. In the current era of COVID-19 vaccination, serological testing will be widely utilized to evaluate the vaccine efficiency. 

Enzyme-linked immunosorbent assay (ELISA), chemiluminescent immunoassay (CLIA), and micro-neutralization (MN) assay are three common serology tests for COVID-19 [14]. MN assay is the gold standard for neutralizing antibody detection. Several in-house and commercial ELISA and CLIA are available, which are based in most cases on SARS-CoV-2 S or NP antigens [14,15,16,17]. Only a few in-house tests were validated against the gold standard MN assay, while the performance of many commercial kits were questionable when evaluated against MN assay [15,16,17].

We previously developed and optimized an S-based ELISA that enables sensitive and specific detection of SARS-CoV-2 IgG antibody in human sera [18]. This protocol was subsequently applied in a number of seroprevalence studies [19,20]. Currently, the number of people who are receiving COVID-19 vaccination is escalating. The most commonly utilized COVID-19 vaccines are based on generating protective neutralizing antibodies to the viral S protein [21]. Hence, in order to distinguish between immunized people due to vaccination from those recovered for the infection, S-based ELISA should be combined with reliable NP-based immunoassays. Moreover, most SARS-CoV-2 new variants carry mutations/deletions in the viral S gene [22,23,24,25,26]. Although there is a lack of conclusive evidence that these mutations/deletions can influence the accuracy of S-based serological testing, this possibility still exists, as their effects on S-based vaccine efficiency were reported [23,24,25,26,27].

In this study, we provide an optimized COVID-19 NP IgG ELISA protocol. The performance of this protocol (sensitivity, specificity, agreement, and overall accuracy) was evaluated against MN assay, in-house S-based ELISA, and Food and Drug Administration (FDA)-approved CLIA. Moreover, the correlation between this indirect NP ELISA with other serological assays was also investigated. Finally, cross-reactivity with antibodies directed against the SARS-CoV-2 S protein or antibodies generated to other coronaviruses (MERS-CoV and HCoV-HKU1) was assessed.

## 2. Materials and Methods

### 2.1. Samples

For optimization of COVID-19 NP IgG ELISA, the number of sero-negative and sero-positive samples utilized in this study was 92 and 90, respectively. Samples were obtained from healthy blood donors and recovered COVID-19 patients. Their serostatus was determined and confirmed by MN assay. Samples were also subjected to previously developed in-house S-Based ELISA and FDA-approved CLIA. Additional samples from individuals who received the S-based COVID-19 vaccination (n = 6) and COVID-19 recovered patients (n = 6) were utilized in order to assess the cross-reactivity with antibodies generated to the SARS-CoV-2 S protein. Human sera containing antibodies to MERS-CoV-2 or HCoV-HKU1 were also used as specificity controls.

### 2.2. Micro-Neutralization (MN) Assay

The sero-status of samples was determined by MN assay conducted as previously described using the local SARS-CoV-2 clinical isolate (SARS-CoV-2/human/SAU/85791C/2020) (Gene accession number MT630432.1) [18]. MN titer of ≥ 1:20 considered positive.

### 2.3. Development and Optimization of COVID-19 NP Human IgG ELISA

The SARS-CoV-2 (2019-nCoV) Nucleocapsid-His recombinant Protein (Sino Biological, Beijing, China) was utilized for ELISA development. Flat Bottom Microtiter plates (SPL Life Sciences) were coated overnight at 4 °C with a range of concentration (typically 6.25 ng to 200 ng per well) of viral recombinant proteins diluted in phosphate buffer saline (PBS). The plates were subsequently washed three times with PBS containing 0.1% Tween 20 (PBST). Blocking buffer (5% skimmed milk in PBST) was added at 100 µL volume per well. The plates were incubated for 1 h at room temperature and then washed three times with PBST. Samples were prepared at a range of dilution (typically 1:100 to 1:3200) in blocking buffer and added at 100 µL volume per well. The plates were incubated for 1 h at 37 °C and then washed six times with PBST. Conjugate (goat KPL peroxidase-labelled antibodies to human IgG; Seracare, Milford, MA, USA) at a dilution of 1:64,000 in PBST was added for an hour at 37 °C. The plates were subjected to six washes with PBST. Finally, 100 µL of 3,3′,5,5′-Tetramethylbenzidine (TMB) (Seracare, Milford, MA, USA) were added for 15 min for color development before stopping the reaction at 100 µL of 1 N hydrochloric acid (HCL). Using Elx 800 bioelisa Reader (Biokit, Barcelona, Spain), the optical density was read at 450 nm (OD_450_). The highest signal to noise ratio for positive controls with minimal background were determined in order to identify the optimized condition.

### 2.4. COVID-19 S-Based IgG ELISA

Testing of human sera for the presence of IgG antibody directed to SARS-CoV-2 S protein was performed using our previously developed in-house S-Based ELISA [18].

### 2.5. COVID-19 S-Based IgG CLIA

Commercially available CLIA (VITROS Immunodiagnostic Products Anti-SARS-CoV-2 IgG Reagent Pack, Reference 619 9919) was used following the manufacturer instructions.

### 2.6. Statistical Analyses

The cut-off value of the developed assay was determined as:

(1) $$\mathrm{Mean}\;\mathrm{values}\;\mathrm{of}\;\mathrm{negative}\;\mathrm{samples}+\left(3*\;\mathrm{standard}\;\mathrm{deviation}\right).$$

The sensitivity, specificity, and agreement were calculated as:

(2) $$\mathrm{Sensitivity}=\left(\frac{\mathrm{The}\;\mathrm{number}\;\mathrm{of}\;\mathrm{true}\;\mathrm{positive}}{\mathrm{The}\;\mathrm{number}\;\mathrm{of}\;\mathrm{true}\;\mathrm{positive}\;\mathrm{plus}\;\mathrm{false}\;\mathrm{negative}}\right)*100$$

(3) $$\mathrm{Specificity}=\left(\frac{\mathrm{The}\;\mathrm{number}\;\mathrm{of}\;\mathrm{true}\;\mathrm{negative}}{\mathrm{The}\;\mathrm{number}\;\mathrm{of}\;\mathrm{true}\;\mathrm{negative}\;\mathrm{plus}\;\mathrm{false}\;\mathrm{positive}}\right)*100$$

(4) $$\mathrm{Agreement}=\left(\frac{\mathrm{The}\;\mathrm{number}\;\mathrm{of}\;\mathrm{true}\;\mathrm{positive}\;\mathrm{and}\;\mathrm{true}\;\mathrm{negative}}{\mathrm{Total}\;\mathrm{number}\;\mathrm{of}\;\mathrm{samples}}\right)*100$$

The receiver-operating characteristic (ROC) was also utilized in order to define the threshold cut-off values that distinguish positive from negative with their corresponding sensitivity and specificity. The correlation between the OD450 values and MN titer was assessed by one-way ANOVA with a *p* value < 0.05 considered statistically significant.

### 2.7. Data Curation

Figure drawing and data processing were performed by GraphPad Prism software.

## 3. Results

### 3.1. Sero-Status of Samples

The samples utilized in this study were human sera collected from healthy blood donors, COVID-19 recovered patients, and COVID-19 vaccinated individuals. In order to determine the sero-status, all samples were initially subjected to the gold standard MN assay with titer of ≥ 1:20 considered positive (data now shown).

### 3.2. Optimization of COVID-19 NP IgG ELISA

**Figure 1:**
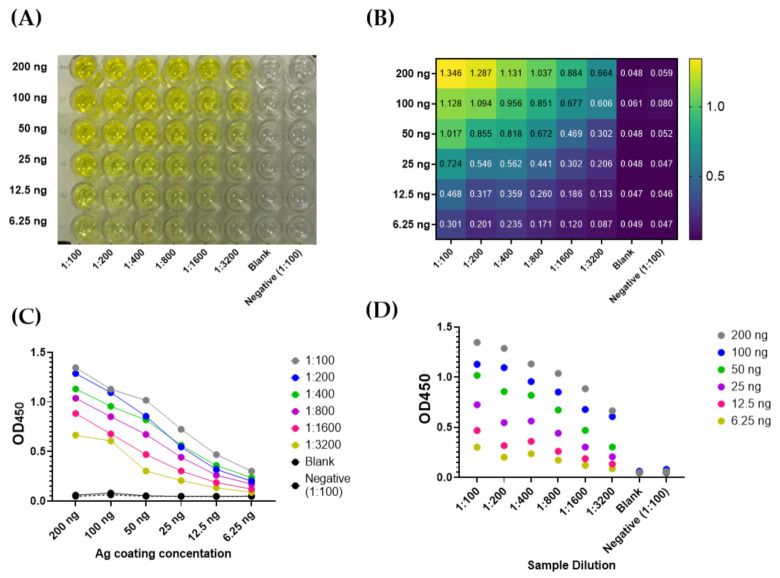
Optimization of an indirect ELISA utilizing SARS-CoV-2 NP recombinant protein. The assay was conducted as described in Section 2.3. (**A**) A representative image of results obtained at a range of SARS-CoV-2 NP coating concentration (6.25 to 200 ng) and a positive sample dilution (1:100 to 1:3200). Negative control and blank were also included. (**B**) Representative OD450 readings. (**C**,**D**) The effect of antigen (Ag) coating concentration and sample dilution on signal readings.

Plates were coated with SARS-CoV-2 NP at a range of concentrations (6.25 ng to 200 ng per well). A serum sample (positive control) was diluted at a range of dilution in blocking buffer (from 1:100 to 1:3200). Conjugate (peroxidase-labelled antibodies to human IgG) was previously optimized and hence, used at a dilution of 1:64,000 in PBST (Figure 1A–D). The highest signal to noise ratio for positive controls with minimal background were chosen as optimized condition, which was as follow: 200 ng/well antigen coating, 1:100 sample dilution, and 1:64,000 conjugate dilution. All subsequent experiments were conducted utilizing this optimized condition.

### 3.3. Cut-Off Value and Assay Validation

**Figure 2:**
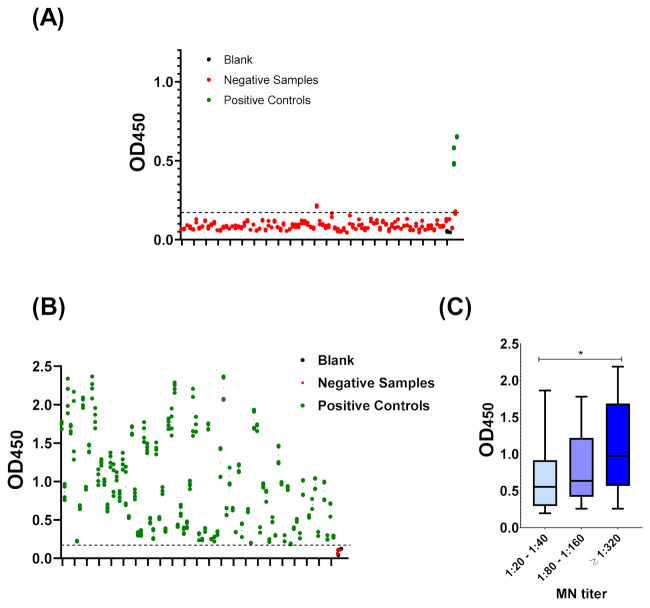
Validation of the developed COVID-19 NP IgG ELISA. (**A**) The cut-off value of the assay. Negative samples (red) and positive control (green) based on micro-neutralization assay in addition to blank (black) were utilized. The actual OD_450_ values for each sample are shown. Dashed lines represent the cut-off value 0.17, which was calculated as mean + (3 × standard deviation). (**B**) Positive samples (green) and negative control (green) based on micro-neutralization assay in addition to blank (black) were utilized. OD_450_ values for all positive samples were above the cut-off value. (**C**) correlation between ELISA results and MN titer. One-way ANOVA was applied. * indicates *p* value < 0.05.

**Figure 3:**
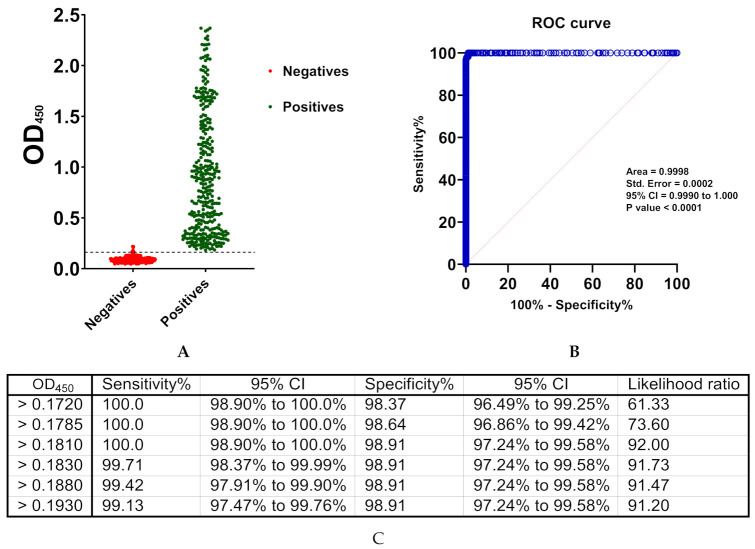
Receiver operating characteristics (ROC) analysis. (**A**) Data utilized for ROC analysis. (**B**) ROC curve. (**C**) A range of cut-off values with their associated sensitivity, specificity, and 95% confidence interval (CI) are shown.

The cut-off value was determined as mean OD_450_ values of 92 negative samples + (3 × standard deviation). Negative samples belonged to healthy blood donors who were not previously diagnosed with COVID-19. Furthermore, their sero-negative status was confirmed by MN assay. The cut-off value of this developed ELISA was 0.17. All OD_450_ values of all negative samples were below 0.17 with an exception of a single sample (Figure 2A). The assay offers 98.9% specificity, which was calculated as described (2.5. Statistical Analyses). On the other hand, the OD_450_ values of all sero-positive samples belonged to COVID-19 recovered patients and confirmed by MN assay were above 0.17 (Figure 2B). Utilizing the described equation (Section 2.6), the sensitivity of the assay was determined as 100% with 98.9% agreement. Although the developed assay should be considered for qualitative applications, a statistically significant correlation with MN titer was observed (Figure 2C). Consistent with the manual calculations, ROC analysis demonstrated an OD_450_ value of 0.181, as the threshold value distinguishes between positive and negative controls while providing maximum sensitivity (100%) and specificity (98.9%) (Figure 3). ROC also demonstrate an overall high accuracy with area under curve (AUC) equal to 0.9998 ± 0.0002; 95% confidence interval (CI) of 0.99 to 1.00. Coefficient of variation (CV) of inter-assay and intra-assay demonstrated high reproducibility with <10% variation (Figure 3).

### 3.4. Compairson with SARS-CoV-2 S-Based ELISA and CLIA

**Figure 4:**
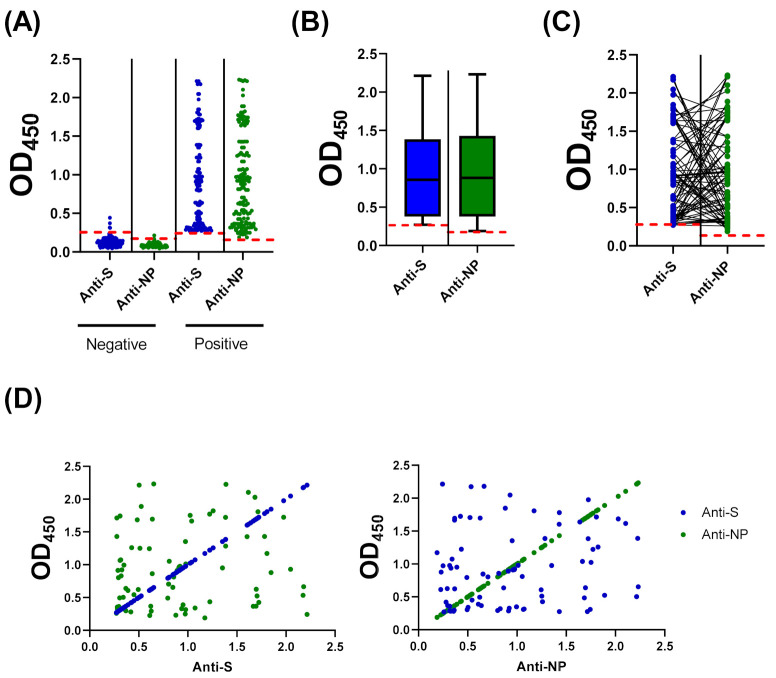
Compairson and correlation between COVID-19 NP and S IgG ELISAs. (**A**) Optical density values at 450 nm (OD_450_) for all negative and positive samples by MN assay using previously develpoed S-based ELISA and the NP-based ELISA protocol optimized in this study. (**B**) Data distribution of positive samples. Boxes: 25th to 75th percentile range; black line: median; whiskers: Min and Max. (**C**) OD_450_ values for each sample as obtained from S- and NP-based ELISAs. (**D**) Non-significant correlation between S- and NP-ELISAs; r^2^ = 0.01849, *p* value = 0.226.

Due to the possbile cross-reactivity of MN assay with neurtlaizaing antibodies from other coronaviruses, we have next compared the results of our optmized NP-based ELISA protocol with our previuosly develpoed S-based ELISA [18]. Among the 92 negative samples, three samples tested positive on the S-based assay (Cut-off OD_450_ value = 0.27). Importantly, all positive samples by MN assay and NP-based ELISA also tested positive by S-based ELISA, which demonstrates concurdance between the three seroligcal assays (Figure 4A). Similar distribution of data was observed when the 25th to 75th percentile range of OD_450_ values were plotted (Figure 4B). However, the OD_450_ value on NP-based ELISA did not correspond to its value on S-based assay (Figure 4C). Indeed, statistical analysis did not find a correlation between the corresponding values obtained from these two assays with r^2^ = 0.01849 and *p* value = 0.226 (Figure 4D).

**Figure 5:**
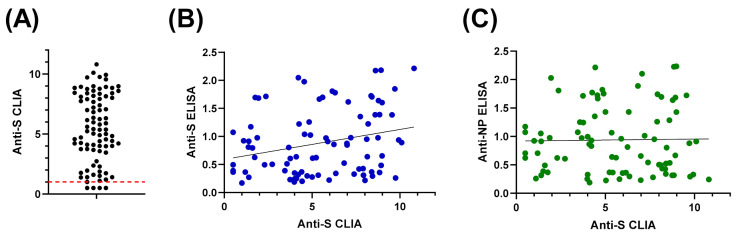
Compairson and correaltion of COVID-19 NP and S IgG ELISAs with FDA-approved CLIA. (**A**) The result obtained from CLIA for all samples that were tested positive by MN assay, S- and NP-based ELISAs. (**B**) Signficant correlation between CLIA and S-based ELISA; r^2^ = 0.2595 and *p* value = 0.015. (**C**) Lack of correlation between CLIA and NP-based ELISA; r^2^ = 0.0167 and *p* value = 0.879.

The 90 samples of recovered COVID-19 patietns that tested positive by MN assay and NP- and S-based ELISAs were also subjected to an FDA-approved CLIA. Most samples (n = 86) tested “reactive”, which validates results obtained from our developed assays (Figure 5A). With regards to the four samples that tested negative, it is highly likely that these results are false-negative, taken into consideration the reported sensitivity of this CLIA [28]. Statistical analyses demonstrated a correlation between CLIA data and S-based ELISA, but not with NP-based assay (Figure 5B,C, respectively).

### 3.5. Evaluation of Assay Cross-Reactivity

Cross-reactivity evaluation was conducted in order to assure that our developed assay specifically detects antibodies directed against SARS-CoV-2. Samples containing antibodies to SARS-CoV-2, MERS-CoV, or HCoV-HKU1, in addition to negative controls and blank, were subjected to the optimized COVID-19 NP IgG ELISA protocol. Among these, only SARS-CoV-2 IgG-containing samples tested positive, excluding cross-reactivity with the other coronaviruses mentioned above.

Next, we assessed the specificity of the developed assay to detect SARS-CoV-2 IgG antibodies directed to the NP protein versus S protein. To achieve this, samples obtained from individuals received the two doses of S-based vaccination versus COVID-19 recovered patients were subjected to the developed NP-based ELISA and the previously reported S-based ELISA [18]. While recovered patients tested positive in both assays, vaccinated people tested positive in the S-based ELISA only.

## 4. Discussion

COVID-19 remains a major public health emergency. Active screening for novel and efficient antivirals, continuous surveillance of new variants, mass COVID-19 vaccination, and evaluation of the seroprevalence status of populations all are key to cope with the pandemic over the next few months [2,13,29,30]. Several reports demonstrated the emergence of new SARS-CoV-2 variants carrying mutations mainly in the viral S gene/protein [22,25,31]. Recent evidences proposed resistance of some of these variants to S-based vaccine-induced antibodies [23,25,26]. Furthermore, accumulation of a mutation on the S gene/protein may affect the performance of some laboratory assays that specifically target this region [27]. With regards to molecular techniques, the current protocol in many countries involve multiple viral gene targets, and therefore the impact of these mutations on the diagnosis might not be crucial. On the other hand, most serology testing target the S protein due to its enhanced antigenicity [32,33], which might compromise the performance of these assays because of the emerging variants. Besides, a systematic review and meta-analysis of the performance of several commercial and in-house SARS-CoV-2 antibody tests revealed sensitivity between 66.7% and 97.9% while specificity ranged from 88.8% to 100% [33]. Some commercial NP-based immunoassays exist, but their validation against MN assay also raised some concerns [15,33]. Indeed, the sensitivity and specificity of these kits sometimes are as low as 81% and 85%, respectively [15,34,35]. Therefore, there is a demand for reliable immunoassays that utilize viral proteins other than S.

Herein, we described a simple qualitative ELISA protocol for detection of SARS-CoV-2 IgG specifically raised to the viral NP. Sufficient antigen concentration that enables efficient capture of the antibody with minimal non-specific backgrounds represents a key element for ELISA optimization. The optimized condition involves antigen coating with 200 ng/well of SARS-CoV-2 NP recombinant protein, sample dilution in blocking buffer at 1:100, and conjugate dilution at 1:64,000 in PBST (Figure 1). Concordance between manual statistical analysis and ROC analysis was observed with a cut-off value of 0.17 (Figure 2 and Figure 3). The assay provides 100% sensitivity (no false negative), 98.8% specificity (minimal false positive), 98.8% agreement, high reproducibility (CV < 10%) and accuracy (AUC = 0.9998 ± 0.0002; 95% CI = 0.99 to 1.00) when evaluated against the gold standard MN assay (Figure 2 and Figure 3). Moreover, a correlation between OD_450_ values and MN titer was observed (Figure 2C). It is crucial to validate ELISA with virus neutralization analysis to conclude the immune protection status confidently [35,36]. Comparative analysis of the optimized NP-based ELISA with our previously developed S-based ELISA and FDA-approved CLIA demonstrated concordant results among these assays (Figure 4 and Figure 5) [18]. Only three samples that tested negative by NP-based ELISA and MN assay were tested positive by S-based assay (Figure 4A). These samples may contain IgG antibodies directed to the SARS-CoV-2 S protein, but they do not confer neutralizing activity. Alternatively, the results of these samples might be interpreted as false positive, taking into consideration of their MN results and the assay specificity (98.4%) [18]. Importantly, all positive samples by NP-based ELISA also tested positive by S-based ELISA with similar data distribution (Figure 4A,B). However, there is lack of positive correlation between OD_450_ values obtained from these ELISAs (Figure 4C,D). The level of antibodies to different viral antigens does not necessary correlate [37,38]. Thus, the lack of correlation between results obtained from NP- and S-based ELISAs might reflect differences in the expression level of the corresponding antibodies. Positive samples (n = 90) by NP- and S-based ELISAs and MN assay were subjected to FDA-approved CLIA. Only four samples were misdiagnosed as false negative by the commercial CLIA, which again raises concern about the performance of COVID-19 commercial serological assays (Figure 5). Indeed, the manufacturer reported 90% sensitivity of this assay, although an independent evaluation estimated it to be 77.4% [28]. 

**Figure 6:**
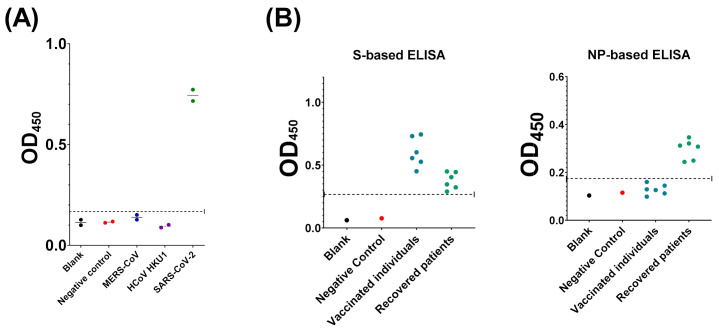
Evaluation of cross-reactivity. (**A**) Human sera containing IgG antibodies to SARS-CoV-2 (green), MERS-CoV (blue), or HCoV-HKU1 (purple) were subjected to the developed COVID-19 NP IgG ELISA. (**B**) Samples obtained for vaccinated people (blue) or recovered patients (green) were tested for the presence of IgG antibodies directed to SARS-CoV-2 S protein (left panel) and NP protein (right panel). Negative control (red) and blank (black) were included in all experiments. Dashed lines represent the assay cut-off value.

Cross-reactivity evaluation is key for assay validation. Herein, we demonstrated a lack of cross-reactivity with anti-MERS-CoV and anti-HCoV-HKU1 antibodies (Figure 6A). Although important, cross-reactivity with other coronaviruses was not assessed due to lack of samples. We also assessed the cross-reactivity of our developed NP-based ELISA with SARS-CoV-2 anti-S antibodies. Recovered patients tested positive in both NP- and S-based ELISAs. On the other hand, participants who received S-dependent vaccines tested positive only in the S-based assay (Figure 6B). These data demonstrate a lack of cross-reactivity with IgG directed to the SARS-CoV-2 S protein. Importantly, the data highlight the significance of utilizing both assays in future seroprevalence studies to distinguish between immunization due to past-infection and vaccination. The reason for the minimal false positive results obtained in our study remains unclear but can be due to interfering substances in the sera or unspecific antigen-antibody interactions.

The ongoing pandemic necessitates improvements in diagnostic test preparedness [34]. In addition to test reliability, affordability is key at this stage due to the economic impact of COVID-19 on healthcare systems [34,39]. In this study, we provided a small-scale laboratory validation of robust COVID-19 anti-NP IgG ELISA. This test platform is known to be compatible with large-scale industrial production [40,41]. It can also be adapted to enable pool testing, which minimizes both cost and time for sample processing [40,41]. However, proper optimization and validation under large-scale pooling conditions are required to maintain the assay reliability.

## 5. Conclusions

The described COVID-19 NP human IgG ELISA protocol is a valuable tool with various applications. It can complement molecular techniques for COVID-19 diagnosis. It also has an important utility in seroprevalence and epidemiological studies such as monitoring the level of herd immunity among communities. Along with S-based ELISA, this NP-based ELISA can be applied to distinguish between individuals who acquired immunity through past infection from those who acquired it from vaccination.

## Data Availability

All data that support the findings of this study are included in this manuscript.

## References

[1] Zhu N., Zhang D., Wang W., Li X., Yang B., Song J., Zhao X., Huang B., Shi W., Lu R. (2020). A novel coronavirus from patients with Pneumonia in China, 2019. N. Engl. J. Med..

[2] Jin Y., Yang H., Ji W., Wu W., Chen S., Zhang W., Duan G. (2020). Virology, epidemiology, pathogenesis, and control of COVID-19. Viruses.

[3] Wu F., Zhao S., Yu B., Chen Y.-M., Wang W., Song Z.-G., Hu Y., Tao Z.-W., Tian J.-H., Pei Y.-Y. (2020). A new coronavirus associated with human respiratory disease in China. Nature.

[4] Ren L.L., Wang Y.M., Wu Z.Q., Xiang Z.C., Guo L., Xu T., Jiang Y.Z., Xiong Y., Li Y.J., Li X.W. (2020). Identification of a novel coronavirus causing severe pneumonia in human: A descriptive study. Chin. Med J..

[5] Wu D., Wu T., Liu Q., Yang Z. (2020). The SARS-CoV-2 outbreak: What we know. Int. J. Infect. Dis. IJID.

[6] Zou X., Chen K., Zou J., Han P., Hao J., Han Z. (2020). Single-cell RNA-seq data analysis on the receptor ACE2 expression reveals the potential risk of different human organs vulnerable to 2019-nCoV infection. Front. Med..

[7] Xiao F., Tang M., Zheng X., Liu Y., Li X., Shan H. (2020). Evidence for gastrointestinal infection of SARS-CoV-2. Gastroenterology.

[8] Cheng Y., Luo R., Wang K., Zhang M., Wang Z., Dong L., Li J., Yao Y., Ge S., Xu G. (2020). Kidney disease is associated with in-hospital death of patients with COVID-19. Kidney Int..

[9] Guan G.W., Gao L., Wang J.W., Wen X.J., Mao T.H., Peng S.W., Zhang T., Chen X.M., Lu F.M. (2020). Exploring the mechanism of liver enzyme abnormalities in patients with novel coronavirus-infected pneumonia. Zhonghua Gan Zang Bing Za Zhi.

[10] Fan C., Li K., Ding Y., Lu W.L., Wang J. (2020). ACE2 expression in kidney and testis may cause kidney and testis damage after 2019-nCoV infection. medRxiv.

[11] Li F., Li W., Farzan M., Harrison S.C. (2005). Structure of SARS coronavirus spike receptor-binding domain complexed with receptor. Science.

[12] Belouzard S., Chu V.C., Whittaker G.R. (2009). Activation of the SARS coronavirus spike protein via sequential proteolytic cleavage at two distinct sites. Proc. Natl. Acad. Sci. USA.

[13] Peeling R.W., Wedderburn C.J., Garcia P.J., Boeras D., Fongwen N., Nkengasong J., Sall A., Tanuri A., Heymann D.L. (2020). Serology testing in the COVID-19 pandemic response. Lancet. Infect. Dis..

[14] Pascarella G., Strumia A., Piliego C., Bruno F., Del Buono R., Costa F., Scarlata S., Agrò F.E. (2020). COVID-19 diagnosis and management: A comprehensive review. J. Intern. Med..

[15] GeurtsvanKessel C.H., Okba N.M.A., Igloi Z., Bogers S., Embregts C.W.E., Laksono B.M., Leijten L., Rokx C., Rijnders B., Rahamat-Langendoen J. (2020). An evaluation of COVID-19 serological assays informs future diagnostics and exposure assessment. Nat. Commun..

[16] Walker G.J., Naing Z., Ospina Stella A., Yeang M., Caguicla J., Ramachandran V., Isaacs S.R., Agapiou D., Bull R.A., Stelzer-Braid S. (2021). SARS Coronavirus-2 Microneutralisation and Commercial Serological Assays Correlated Closely for Some but Not All Enzyme Immunoassays. Viruses.

[17] Jääskeläinen A.J., Kuivanen S., Kekäläinen E., Ahava M.J., Loginov R., Kallio-Kokko H., Vapalahti O., Jarva H., Kurkela S., Lappalainen M. (2020). Performance of six SARS-CoV-2 immunoassays in comparison with microneutralisation. J. Clin. Virol..

[18] Alandijany T.A., El-Kafrawy S.A., Tolah A.M., Sohrab S.S., Faizo A.A., Hassan A.M., Alsubhi T.L., Othman N.A., Azhar E.I. (2020). Development and optimization of in-house ELISA for detection of human IgG antibody to SARS-CoV-2 full length spike protein. Pathogens.

[19] Alandijany T.A., El-Kafrawy S.A., Al-Ghamdi A.A., Qashqari F.S., Faizo A.A., Tolah A.M., Hassan A.M., Sohrab S.S., Hindawi S.I., Badawi M.A. (2021). Lack of Antibodies to SARS-CoV-2 among Blood Donors during COVID-19 Lockdown: A Study from Saudi Arabia. Healthcare.

[20] Ahmed W.A., Dada A., Alshukairi A.N., Sohrab S.S., Faizo A.A., Tolah A.M., El-Kafrawy S.A., Bajrai L.H., Moalim H.M., Aly M.H. (2021). Seroprevalence of neutralizing antibodies to severe acute respiratory syndrome coronavirus 2 (SARS-CoV-2) among healthcare workers in Makkah, Saudi Arabia. J. King Saud Univ. Sci..

[21] Kashte S., Gulbake A., El-Amin Iii S.F., Gupta A. (2021). COVID-19 vaccines: Rapid development, implications, challenges and future prospects. Hum. Cell.

[22] Tegally H., Wilkinson E., Lessells R.J., Giandhari J., Pillay S., Msomi N., Mlisana K., Bhiman J.N., von Gottberg A., Walaza S. (2021). Sixteen novel lineages of SARS-CoV-2 in South Africa. Nat. Med..

[23] Chen R.E., Zhang X., Case J.B., Winkler E.S., Liu Y., VanBlargan L.A., Liu J., Errico J.M., Xie X., Suryadevara N. (2021). Resistance of SARS-CoV-2 variants to neutralization by monoclonal and serum-derived polyclonal antibodies. Nat. Med..

[24] Weisblum Y., Schmidt F., Zhang F., DaSilva J., Poston D., Lorenzi J.C., Muecksch F., Rutkowska M., Hoffmann H.H., Michailidis E. (2020). Escape from neutralizing antibodies by SARS-CoV-2 spike protein variants. eLife.

[25] Rees-Spear C., Muir L., Griffith S.A., Heaney J., Aldon Y., Snitselaar J.L., Thomas P., Graham C., Seow J., Lee N. (2021). The effect of spike mutations on SARS-CoV-2 neutralization. Cell Rep..

[26] Wang P., Nair M.S., Liu L., Iketani S., Luo Y., Guo Y., Wang M., Yu J., Zhang B., Kwong P.D. (2021). Antibody resistance of SARS-CoV-2 Variants B.1.351 and B.1.1.7. Nature.

[27] Pereira F. (2021). SARS-CoV-2 variants lacking a functional ORF8 may reduce accuracy of serological testing. J. Immunol. Methods.

[28] Public Health England (2020). Evaluation of the Ortho Clinical Diagnostics Vitros Immunodiagnostic Products Anti-SARS-CoV-2 IgG Serology Assay for the Detection of Anti-SARS-CoV-2 Antibodies.

[29] Alandijany T.A., Faizo A.A., Azhar E.I. (2020). Coronavirus disease of 2019 (COVID-19) in the Gulf Cooperation Council (GCC) countries: Current status and management practices. J. Infect. Public Health.

[30] Peto J. (2020). Covid-19 mass testing facilities could end the epidemic rapidly. BMJ.

[31] Altmann D.M., Boyton R.J., Beale R. (2021). Immunity to SARS-CoV-2 variants of concern. Science.

[32] Jacofsky D., Jacofsky E.M., Jacofsky M. (2020). Understanding Antibody Testing for COVID-19. J. Arthroplast..

[33] Lisboa Bastos M., Tavaziva G., Abidi S.K., Campbell J.R., Haraoui L.-P., Johnston J.C., Lan Z., Law S., MacLean E., Trajman A. (2020). Diagnostic accuracy of serological tests for covid-19: Systematic review and meta-analysis. BMJ.

[34] Vandenberg O., Martiny D., Rochas O., van Belkum A., Kozlakidis Z. (2021). Considerations for diagnostic COVID-19 tests. Nat. Rev. Microbiol..

[35] Rathe J.A., Hemann E.A., Eggenberger J., Li Z., Knoll M.L., Stokes C., Hsiang T.-Y., Netland J., Takehara K.K., Pepper M. (2020). SARS-CoV-2 serologic assays in control and unknown populations demonstrate the necessity of virus neutralization testing. J. Infect. Dis..

[36] Luchsinger L.L., Ransegnola B.P., Jin D.K., Muecksch F., Weisblum Y., Bao W., George P.J., Rodriguez M., Tricoche N., Schmidt F. (2020). Serological assays estimate highly variable SARS-CoV-2 neutralizing antibody activity in recovered COVID-19 patients. J. Clin. Microbiol..

[37] Murrell I., Forde D., Zelek W., Tyson L., Chichester L., Palmer N., Jones R., Morgan B.P., Moore C. (2021). Temporal development and neutralising potential of antibodies against SARS-CoV-2 in hospitalised COVID-19 patients: An observational cohort study. PLoS ONE.

[38] Sun B., Feng Y., Mo X., Zheng P., Wang Q., Li P., Peng P., Liu X., Chen Z., Huang H. (2020). Kinetics of SARS-CoV-2 specific IgM and IgG responses in COVID-19 patients. Emerg. Microbes Infect..

[39] Kaye A.D., Okeagu C.N., Pham A.D., Silva R.A., Hurley J.J., Arron B.L., Sarfraz N., Lee H.N., Ghali G.E., Gamble J.W. (2020). Economic impact of COVID-19 pandemic on healthcare facilities and systems: International perspectives. Best Pract. Res. Clin. Anaesthesiol..

[40] Allen J.W.L., Verkerke H., Owens J., Saeedi B., Boyer D., Shin S., Roback J.D., Neish A.S., Stowell S.R. (2021). Serum pooling for rapid expansion of anti-SARS-CoV-2 antibody testing capacity. Transfus. Clin. Biol..

[41] Cerutti H., Ricci V., Tesi G., Soldatini C., Castria M., Vaccaro M.N., Tornesi S., Toppi S., Verdiani S., Brogi A. (2021). Large scale production and characterization of SARS-CoV-2 whole antigen for serological test development. J. Clin. Lab. Anal..

